# Melatonin Treatment Triggers Metabolic and Intracellular pH Imbalance in Glioblastoma

**DOI:** 10.3390/cells11213467

**Published:** 2022-11-02

**Authors:** Beatriz I. Fernandez-Gil, Andrea Otamendi-Lopez, Alexandra Bechtle, Carla A. Vazquez-Ramos, Neda Qosja, Paola Suarez-Meade, Rachel Sarabia-Estrada, Mark E. Jentoft, Hugo Guerrero-Cázares, Germaine Escames, Paula Schiapparelli, Alfredo Quiñones-Hinojosa

**Affiliations:** 1 Department of Neurologic Surgery, Mayo Clinic, 4500 San Pablo Road, Jacksonville, FL 32224, USA; 2 Department of Laboratory Medicine and Pathology, Mayo Clinic, Jacksonville, FL 32224, USA; 3 Instituto de Biotecnología Centro de Investigación Biomédica, Universidad de Granada, 18016 Granada, Spain

**Keywords:** intracellular acidity, cancer metabolism, lactate, MCT4, ROS, LDHA, GBM, OXPHOS, glycolysis

## Abstract

Metabolic rewiring in glioblastoma (GBM) is linked to intra- and extracellular pH regulation. In this study, we sought to characterize the role of melatonin on intracellular pH modulation and metabolic consequences to identify the mechanisms of action underlying melatonin oncostatic effects on GBM tumor initiating cells. GBM tumor initiating cells were treated at different times with melatonin (1.5 and 3.0 mM). We analyzed melatonin’s functional effects on GBM proliferation, cell cycle, viability, stemness, and chemo-radiosensitivity. We then assessed the effects of melatonin on GBM metabolism by analyzing the mitochondrial and glycolytic parameters. We also measured the intracellular and extracellular pH. Finally, we tested the effects of melatonin on a mouse subcutaneous xenograft model. We found that melatonin downregulated LDHA and MCT4, decreasing lactate production and inducing a decrease in intracellular pH that was associated with an increase in ROS and ATP depletion. These changes blocked cell cycle progression and induced cellular death and we observed similar results in vivo. Melatonin’s cytotoxic effects on GBM were due, at least in part, to intracellular pH modulation, which has emerged as a newly identified mechanism, providing new insights into the oncostatic effect of melatonin on GBM.

## 1. Introduction

Glioblastoma (GBM) is the most common and lethal primary brain tumor in adults. The lack of long-term, effective therapy merits ongoing research to identify novel approaches.

In GBM, metabolic rewiring from oxidative phosphorylation (OXPHOS) toward a more-glycolytic state drives tumor aggressiveness and resistance to treatment [1]. These metabolic adaptations are linked to changes in extracellular and intracellular pH, and the more-acidic extracellular microenvironment and more-alkaline intracellular milieu are considered hallmarks of cancer [2]. High intracellular pH supports escape from apoptosis, promotes proliferation, and increases the resistance to radio and chemotherapy [3]. The acidic extracellular environment enables migration and invasion via extracellular matrix degradation, and it also promotes vascularization and immune suppression. Thus, slight variations in pH homeostasis are likely to be important for tumor survival [3,4,5,6], and the characterization of pH regulation in GBM merits a deeper look.

N-Acetyl-5-methoxytryptamine (aMT; melatonin) is a versatile and pleiotropic indolamine. The role of melatonin in cancer has been studied in recent years because of its oncostatic effects [7,8]. Despite the well-known effects of aMT as an antioxidant [9], our team and others have described the role of melatonin as an inducer of reactive oxygen species (ROS) [10,11] in different cancer types at pharmacological concentrations [12,13]. Although ROS are widely believed to directly trigger apoptotic mechanisms, controversy remains about how these free radicals are generated and if they are the only mechanism underlying the anticancer effects of melatonin. Little research has focused on the regulatory effects of melatonin on the intracellular pH of cancer cells [14]. Here, we describe for the first time the modulatory effects of melatonin on GBM intracellular pH and consider the implications of these findings on cancer metabolism and the fate of GBM cells.

## 2. Materials and Methods

### 2.1. Cell Culture and Reagents

We used five glioma tumor initiating cell lines (GBM1A [15], QNS120, GBM612, GBM965, and QNS108 [16]) that were derived from intraoperative tissue samples of patients with GBM (Table 1). Patients were newly diagnosed and had not received prior treatment. Cells were cultured as described previously [16,17].

aMT stock solution (800013; Fagron, St Paul, MN, USA) was dissolved in 15% propylene glycol (81172-1L, Sigma-Aldrich, St. Louis, MO, USA) in PBS, as previously described [13,18]. Cells were treated with the vehicle or with aMT (1.0, 1.5, 3.0, 5.0 mM). We treated cells with agomelatine (S1243; Selleck Chemicals, Houston, TX, USA) or tasimelteon (S4281; Selleck Chemicals) (0.1, 0.25, 0.5, 1.0 mM) dissolved in dimethyl sulfoxide (D8418-500ML; Sigma-Aldrich) for 48 h. We also treated cells with temozolomide (TMZ) (S1237, Selleck Chemicals) for 48 h (0.7 and 3.0 mM), either alone or after a 48-h pretreatment with melatonin or the vehicle. For radiation treatment, cells pretreated with aMT or the vehicle for 48 h were exposed to radiation doses of 0.5, 1.0, or 1.5 Gy (XRad160 biological irradiator; Precision X-Ray, Inc., St. Louis, North Branford, CT, USA).

### 2.2. Cell Proliferation and Viability

We quantified cell proliferation after treating cells for 48 h with aMT (as described above). We used the CyQUANT Cell Proliferation Assay Kit (C35011; Invitrogen, Waltham, MA, USA) following the manufacturer’s instructions. Fluorescence was measured with an HTX Synergy microplate reader (BioTek Instruments, Inc., Winooski, VT, USA) with an excitation wavelength of 480 nm and an emission wavelength of 520 nm. We measured the viability of cells treated with aMT combined with TMZ by using the MTT (3-[4,5-dimethylthiazol-2-yl]-2,5-diphenyltetrazolium bromide) assay (V131514; Thermo Fisher Scientific, Waltham, MA, USA), following the manufacturer’s instructions. Optical density was measured at 570 nm in a HTX Synergy microplate reader (BioTek Instruments, Inc.).

### 2.3. Cell Cycle Assay

DNA content was assessed after 48 h of treatment through flow cytometry and the Click-iT EdU Cell Proliferation Kit (C10337; Thermo Fisher Scientific), in accordance with the manufacturer’s instructions. Samples were run in a CytoFLEX cytometer (Beckman Coulter, Indianapolis, IN, USA). Data were reported as the percentage of cells in each phase of the cell cycle.

### 2.4. Apoptosis

Apoptosis was measured by quantifying activated caspase-3 and caspase-7 (caspase-3/7) with the CellEvent Caspase-3/7 Green Detection Reagent (C10423; Thermo Fisher Scientific), following the manufacturer’s instructions. Fluorescence was measured in a SpectraMax M5 microplate reader (Molecular Devices, LLC. San Jose, CA, USA) with the following excitation/emission wavelengths: activated caspase-3/7, 502/530 nm; ethidium homodimer, 530/640 nm; and Hoechst, 350/452 nm.

### 2.5. Clonogenic Assay

Cells were treated with melatonin alone or melatonin plus radiation, as described above. After 2 weeks, colonies were fixed with 100% ethanol and then stained with 2.3% crystal violet. Colonies of at least 50 cells were counted with ImageJ software (NIH, Bethesda, MD, USA).

### 2.6. Synergy Calculation

The degree of synergy was calculated with the reference model highest single agent (HSA) using the software SynergyFinder 2.0: visual analytics of multidrug combination synergies [19]. Synergy scores < −10 indicate antagonism, from −10 to 10 addition, and >10 synergism.

### 2.7. Limiting Dilution Assay

The limiting dilution assay was performed as previously described [17]. Briefly, the day after seeding, cells were treated with the vehicle or melatonin (1.5 or 3.0 mM). Sphere formation was monitored for 12 days. Results were analyzed using the Extreme Limiting Dilution Analysis online software tool [20].

### 2.8. Oxygen Consumption Rate

The oxygen consumption rate (OCR) was determined with the Seahorse XF Cell Mito Stress Test Kit (103015-100; Agilent Technologies, Santa Clara, CA, USA) and the extracellular Flux analyzer (XFe96 Seahorse analyzer; Agilent Technologies) by following the manufacturer’s instructions. Briefly, cells treated with the vehicle or melatonin underwent three sequential injections of oligomycin, FCCP, and rotenone/antimycin A, after recording the basal measurements. The OCR values were normalized to 10^4^ cells with the CyQUANT Direct Cell Proliferation Assay (C35011; Invitrogen). Data shown are the most representative of three biological replicates.

### 2.9. Pyruvate Concentration

Intracellular pyruvate concentration was fluorimetrically measured by using the EnzyChrom Pyruvate Assay Kit (EPYR-100; BioAssay Systems, Hayward, CA, USA) in a microplate reader (BioTek Instruments, Inc.) with an excitation wavelength of 530 nm and an emission wavelength of 585 nm, following the manufacturer’s instructions. Data were normalized to the protein content, as measured with the bicinchoninic acid (BCA) protein assay (23227; Thermo Fisher Scientific).

### 2.10. Western Blotting

Western blotting was performed as previously described [17]. We used the following primary antibodies: MCT4 (A304-439A; Bethyl Laboratories, Montgomery, TX, USA); LDHA (3582s; Cell Signaling Technology, Danvers, MA, USA); PDHA (3205S; Cell Signaling Technology); PKM2 (4053S; Cell Signaling Technology); and GAPDH (sc-47724; Santa Cruz Biotechnology, Inc. Dallas, TX, USA). We used the following secondary antibodies: goat antirabbit (31460; Thermo Fisher Scientific) or goat antimouse IgG (31430; Thermo Fisher Scientific) HRP conjugated. Proteins were visualized with the SuperSignal West Pico PLUS Chemiluminescent Substrate (34580; Thermo Fisher Scientific) by following the manufacturer’s instructions. Images were analyzed with an Amersham Imager 600 (GE Healthcare, Chicago, IL, USA). Protein band intensity was quantified with ImageJ software (National Institutes of Health) and normalized to the total protein that was detected with GelCode Blue Stain Reagent (24590; Thermo Fisher Scientific). Data were expressed relative to the controls.

### 2.11. ROS Production

ROS production was measured using CellROX™ Green Reagent (Invitrogen, C10444). The cell-permeant dye is weakly fluorescent while in a reduced state and exhibits bright green photostable fluorescence upon oxidation by reactive oxygen species (ROS) and subsequent binding to DNA, with absorption/emission maxima of ∼485/520 nm, measured in a SpectraMax M5 microplate reader (Molecular Devices). The ROS levels were measured every 24 h for 4 days in the Operetta CLS High Content Analysis System (Perkin Elmer Inc., Hopkinton, MA, USA) with an excitation wavelength of 490 nm and an emission wavelength of 525 nm. Results were normalized by Hoechst staining.

### 2.12. Glucose-6-Phosphate Dehydrogenase Activity

Glucose-6-phosphate dehydrogenase (G6PDH) activity was performed with the Glucose-6-Phosphate Dehydrogenase Activity Assay Kit (700300; Cayman Chemical, Ann Arbor, MI, USA) by following the manufacturer’s instructions. Fluorescence was measured with an HTX Synergy microplate reader (BioTek Instruments, Inc.) with an excitation wavelength of 530 nm and an emission wavelength of 585 nm. Results were normalized by protein content using the BCA method.

### 2.13. Substrate Oxidation

Cellular substrate oxidation was assessed by measuring the changes in OCR when specifically blocking three of the primary substrates that fuel mitochondria. Etomoxir (103672-100; Agilent Technologies) blocks long-chain fatty acids by inhibiting carnitine palmitoyl transferase 1a; UK5099 (103673-100; Agilent Technologies) blocks pyruvate by inhibiting the mitochondrial pyruvate carrier; and BPTES (103674-100; Agilent Technologies) blocks glutamine by inhibiting glutaminase 1. We performed the assay by following the manufacturer’s instructions and the combined pathway inhibitors with the Seahorse XF Cell Mito Stress Test Kit (103015-100; Agilent Technologies) in a XFe96 Seahorse analyzer (Agilent Technologies), as described above. Data were analyzed with Seahorse Analytics software. Data shown are the most representative of three biological replicates. The OCR values were normalized to 10^4^ cells with the CyQUANT Direct Cell Proliferation Assay.

### 2.14. ATP Detection

ATP was detected with the ATP Determination Kit (A22066; Thermo Fisher Scientific) by following the manufacturer’s instructions. Luminescence was measured with an HTX Synergy microplate reader (BioTek Instruments, Inc.). Results were normalized in mirror wells by Hoechst.

### 2.15. pH

Extracellular pH was measured with a Fisher Scientific Accumet AE150 pH meter. Intracellular pH was quantified using both the pHrodo™ Red AM Intracellular pH Indicator (P35372, Invitrogen) and the Intracellular pH Assay Kit (ab228552; Abcam Inc. Waltham, MA, USA). Assays were performed by following the manufacturer’s instructions. pHrodo Red is barely fluorescent at neutral pH, but increasingly fluorescent as the pH drops. Fluorescence was measured in a SpectraMax M5 microplate reader (Molecular Devices) with excitation/emission of 560/585 nm at 48 and 96 h after melatonin treatment. Then, using the Intracellular pH Calibration Buffer Kit (P35379, Invitrogen), we calculated the absolute pH values. Briefly, pH 6.5 and pH 7.5 buffers were sequentially clamped to intracellular pH using a valinomycin/nigericin solution, and the pHrodo results were calculated from the standard curve. In contrast, the Abcam kit decreases in fluorescence signal after a reduction in intracellular pH. Fluorescence intensity was measured with an HTX Synergy microplate reader (BioTek Instruments, Inc.) with an excitation wavelength of 490 nm and an emission wavelength of 535 nm at a 96 h timepoint.

### 2.16. Lactate Concentration

Lactate concentration was quantified with the EnzyChrom L-Lactate Assay Kit (ECLC-100; Bioassay Systems). Assays were performed by following the manufacturer’s instructions. Optical density was measured at 565 nm in a HTX Synergy microplate reader (BioTek Instruments, Inc.). Results were normalized by protein content using the BCA method.

### 2.17. Glucose Availability

Glucose in the media was measured overtime using the GlucCell Glucose Monitoring System (CLS-1322-02, Chemglass Life Sciences LLC. Vineland, NJ, USA) following the manufacturer’s instructions.

### 2.18. In Vivo Subcutaneous Xenografts

Subcutaneous xenografts were established in the flanks of 14 athymic nude male mice. We injected 4 × 10^6^ GBM1A GFP-Luc+ cells in a 1:1 base media-Matrigel suspension. We evaluated the tumor engraftment with caliper measurements and with bioluminescence (IVIS Spectrum System; Perkin Elmer) using D-luciferin (XenoLight [15 mg/mL]; 122799; Perkin Elmer). When the tumor reached a volume of around 100 mm^3^, we treated mice intratumorally with melatonin 3% (WO2018178497) or the vehicle for 5 days a week for 2 weeks. Tumors were measured every 3 days for 15 days, and bioluminescence imaging (BLI) was performed once a week. Tumor volume was calculated with the formula volume = (length × width^2^)/2. At the end of the treatment period, tumors were extracted, photographed, weighed, and measured.

### 2.19. Histologic Analysis

We performed hematoxylin-eosin staining for the structural detail and immunohistochemistry of Ki67 and caspase-3 to evaluate cell proliferation and apoptosis, respectively. The Masson trichrome stain was applied to visualize the collagen fibers and detect fibrosis. Tumor samples were fixed in 4% paraformaldehyde for 24 h and then dehydrated and paraffinized. Four-micron-thick sections were stained with hematoxylin (22-500-053; Thermo Fisher Scientific) and eosin (22-500-063; Thermo Fisher Scientific). Immunohistochemical staining with Ki67 (ab92742, 1:250; Abcam) and cleaved caspase-3 (9664S, 1:100; Cell Signaling Technology) was performed to evaluate cell proliferation and apoptosis, respectively, and the Masson trichrome stain was applied to visualize the collagen fibers and detect fibrosis. Slides were scanned as high-resolution images with ScanScope (Aperio). The ratio of Ki67-positive nuclei to total cells and the ratio of caspase-3 positive cells to total cells were determined with Image Scope software (Leica Biosystems, Deer Park, IL, USA).

### 2.20. Statistical Analysis

Statistical analyses were performed with Prism 9 scientific software (GraphPad Software Inc., La Jolla, CA, USA). Unpaired 2-tailed Student t tests were used for comparisons of two groups. Multiple-comparison analyses were performed with 1- or 2-way analysis of variance (ANOVA), followed by the Tukey or Dunnett’s correction. Results are shown as the mean ± SEM of three replicates in independent experiments, unless stated otherwise. * *p* < 0.05; ** *p* < 0.01; *** *p* < 0.001.

## 3. Results

### 3.1. Melatonin Decreases GBM Viability and Stemness, Inducing Cell Death

**Figure 1:**
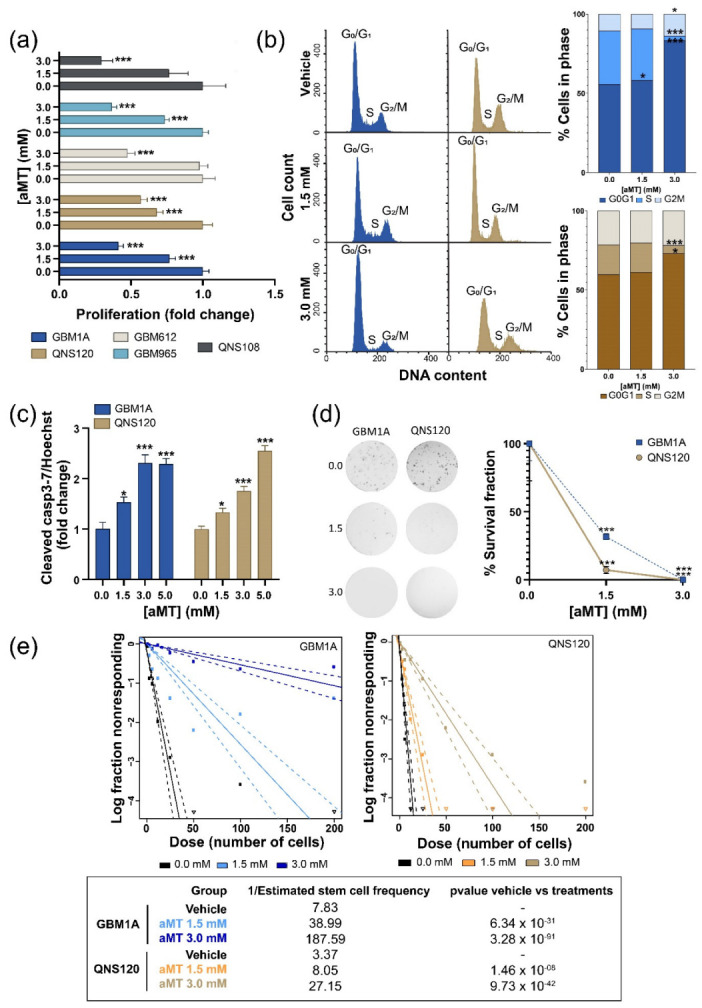
Melatonin (aMT) decreased viability and clonogenic ability, leading to cell cycle arrest and cell death by apoptosis in GBM. (**a**) Proliferation of five patient-derived cell lines after 48 h of treatment with vehicle or aMT. (**b**) Representative histograms showing the distribution of cell cycle phases and quantification of GBM1A and QNS120 after 48 h of treatment with the vehicle or aMT. (**c**) Apoptotic cell death is indicated by the ratio of activated caspase-3/7 over Hoechst. GBM1A and QNS120 were treated for 96 h with the vehicle or aMT. (**d**) Colony-formation assay in GBM1A and QNS120 with vehicle or aMT. Representative colonies are shown; the graph summarizes the survival fraction. (**e**) Limiting dilution assay shows the proportion of nonresponding cells. The graph shows a log-fraction plot (each line represents the log of the active cell fraction; dotted lines represent the 95% CI). * *p* < 0.05; *** *p* < 0.001.

We began by assessing proliferation after melatonin treatment in a panel of primary derived GBM tumor initiating cell lines (Table 1). High concentrations of melatonin significantly reduced proliferation in the five cell lines regardless of their molecular background (Figure 1A). Among the panel of cell lines, we chose GBM1A and QNS120 to explore further because of their basal differences at the metabolic level, with GBM1A more glycolytic while QNS120 was more dependent on mitochondrial respiration as an energy source (Figure S1a). GBM1A and QNS120 were then treated with melatonin agonists agomelatine and tasimelteon and consistently showed diminished proliferation (Figure S1b,c). The decreased proliferation after melatonin treatment was associated with a decrease in the percentage of GBM1A and QNS120 cells in the S phase (vehicle vs. aMT 3.0 mM GBM1A, *p* < 0.001; QNS120, *p* < 0.001), and it was associated with cell cycle arrest at G0/G1 in both GBM1A and QNS120 and G2/M in GBM1A (Figure 1B).

Melatonin also induced an increase in cellular death attributable to programmed cell death, determined by an increased level of cleaved caspase-3/7 (Figure 1c), therefore indicating apoptotic cell death. Moreover, melatonin also reduced the cells’ ability to repopulate and self-renew, as shown by the clonogenic assay (Figure 1d) and the limiting dilution assay (Figure 1e). In addition, we wanted to determine whether melatonin sensitized GBM cells to the standard of care treatments. For this purpose, we performed the HSA synergy analysis using the temozolomide and radiation treatments. We observed that melatonin combined with TMZ (Figure S1d–g) showed a synergistic effect with all combinations in both GBM1A and QNS120, showing HAS synergy scores of 10.81 and 23.43, respectively. When combined with RT (Figure S1h–k), 1.5 mM aMT had the highest synergy scores in both cell lines. However, at 0.75 mM aMT, the synergy decreased. The highest concentration of aMT (3 mM) had a strong effect on the cells and did not allow colony formation.

In summary, melatonin reduced GBM viability by increasing cell death and reducing stemness while enhancing synergism with the current GBM standard of care treatments for both cell lines in vitro.

### 3.2. Melatonin Differentially Disturbs GBM Mitochondrial Metabolism

**Figure 2:**
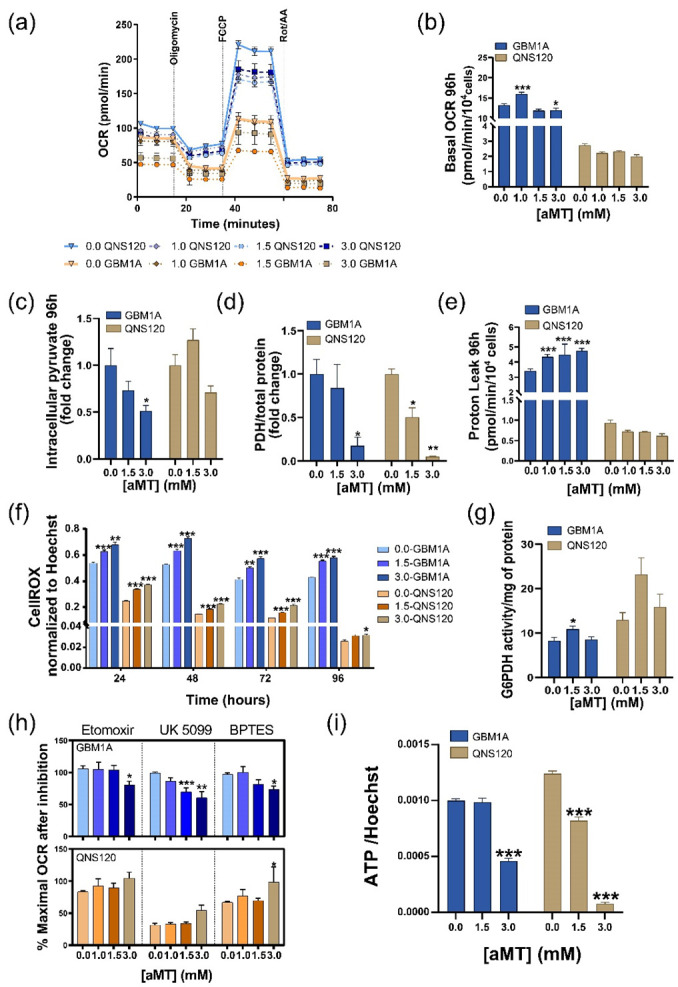
Melatonin (aMT) differentially disturbs GBM mitochondrial metabolism. (**a**) Oxygen consumption rate (OCR) kinetics and (**b**) basal OCR for GBM1A and QNS120 after 96 h of treatment with the vehicle or aMT. (**c**) Intracellular pyruvate in GBM1A and QNS120 after 96 h of treatment with the vehicle or aMT. (**d**) Pyruvate dehydrogenase (PDH) protein relative expression after 96 h of treatment with the vehicle or aMT. (**e**) Proton leak from GBM1A and QNS120 after 96 h of treatment with the vehicle or aMT. (**f**) ROS levels in GBM1A and QNS120 during treatment with the vehicle or aMT. (**g**) Glucose-6-phosphate dehydrogenase (G6PDH) activity in GBM1A after 48 h of treatment with the vehicle or aMT. (**h**) Percentage of maximal OCR after inhibition with etomoxir, UK5099, or BPTES for GBM1A and QNS120 treated for 96 h with the vehicle or aMT. (**i**) ATP depletion in GBM1A and QNS120 after 96 h of treatment with the vehicle or aMT. * *p* < 0.05; ** *p* < 0.01; *** *p* < 0.001.

**Figure 3:**
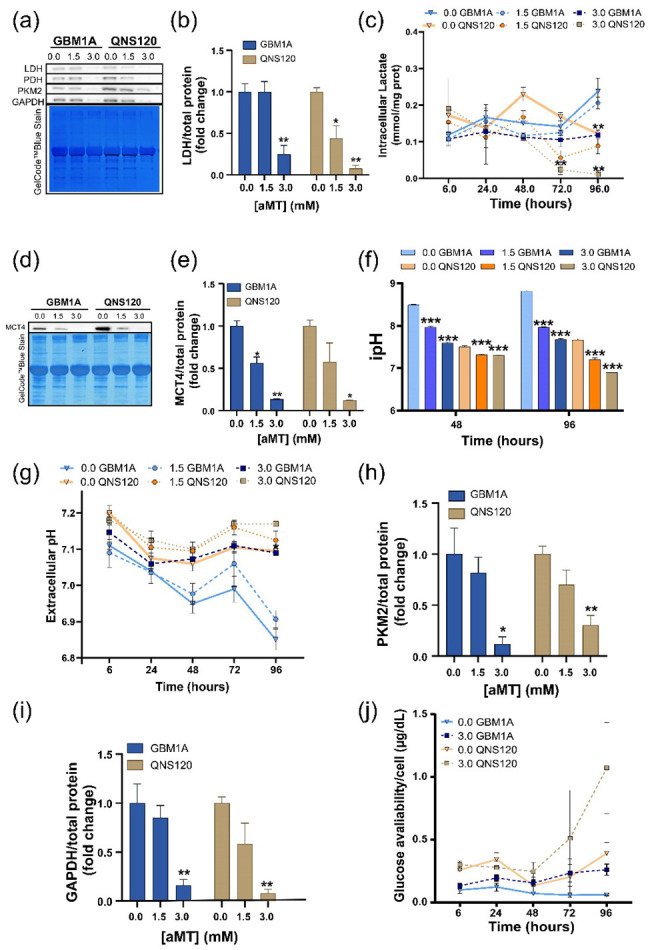
Melatonin (aMT) disrupts the pH balance in GBM while downregulating glycolysis. (**a**) Representative Western blot of lactate dehydrogenase (LDH), pyruvate dehydrogenase (PDH), pyruvate kinase subtype M2 (PKM2), and glyceraldehyde 3-phosphate dehydrogenase (GAPDH) after 96 h of treatment with the vehicle or aMT. (**b**) Western blot quantification of lactate dehydrogenase (LDH) after 96 h of treatment with vehicle or aMT; (**c**) Intracellular lactate overtime after treatment with vehicle or aMT in GBM1A and QNS120; (**d**) Representative Western blot of Monocarboxylate transporter 4 (MCT4) after 96 h of treatment with vehicle or aMT; (**e**) Western blot quantification of MCT4 after 96 h of treatment with vehicle or aMT; (**f**) Intracellular pH after 48 or 96 h with vehicle or aMT in GBM1A; (**g**) Extracellular pH overtime after treatment with vehicle or aMT in GBM1A and QNS120; (**h**) Western blot quantification of pyruvate kinase subtype M2 (PKM2) and (**i**) glyceraldehyde 3-phosphate dehydrogenase (GAPDH) after 96 h of treatment with vehicle or aMT; (**j**) Glucose availability in media overtime in GBM1A and QNS120. * *p* < 0.05; ** *p* < 0.01; *** *p* < 0.001.

After evaluating melatonin’s functional effects across multiple GBM cell lines, we wanted to explore how these effects were related to the role of melatonin on mitochondrial homeostasis. For this purpose, we measured the OCR over a wide range of aMT concentrations (0–3.0 mM). First, we observed that cells treated for 48 h with aMT 1.5 mM increased the basal OCR in GBM1A and QNS120, and 3.0 mM aMT only in GBM1A (Figure S2a,b), correlating with a decrease in pyruvate levels in GBM1A. In contrast, we found an accumulation in pyruvate levels with aMT 3.0 mM in QNS120 at the same timepoint (Figure S2c). At 96 h, we observed that basal OCR increased with aMT 1.0 mM but started to decrease at higher concentrations in GBM1A. In contrast, we did not observe any changes in QNS120 OCR at any concentration at 96 h (Figure 2a,b). Pyruvate at this timepoint slowed down its decrease in GBM1A and stop increasing in QNS120 (Figure 2c). This reduction in OCR levels could correlate to the decrease in pyruvate dehydrogenase (PDH) expression for both cell lines at aMT 3.0 mM (Figure 2e and Figure 3a). Additionally, we evaluated the effects of melatonin on proton leak. We found a significant increase at aMT 1.0, 1.5, and 3.0 for GBM1A. However, we did not find significant differences for QNS120 (Figure 2e). Accordingly, coupling efficiency decreased in GBM1A and remained stable in QNS120 after melatonin treatment (Figure S2d). Proton leak regulates ROS production at the mitochondrial level. Indeed, we showed that GBM1A cells had an increase in ROS that was induced by aMT in a dose- and time-dependent manner (Figure 2f). Surprisingly, QNS120 also presented a strong ROS increase that did not correlate with the proton leak levels. Furthermore, we wanted to see whether the changes in ROS were reflected in the pentose phosphate pathway. We analyzed the effect of melatonin on G6PDH activity and showed that aMT 1.5 mM significantly increased its activity in GBM1A at 96 h and showed a similar trend in QNS120, although this was not significant (Figure 2g).

Since there were metabolic differences between the two cell lines at the baseline, we decided to evaluate cellular substrate oxidation to see whether the dependency on the energy sources were the same as at the basal level. GBM1A showed strong dependency in mitochondrial respiration after treatment with aMT 3.0 mM, showing a reduction in the maximal OCR levels, especially after pyruvate carrier inhibition with UK 5099, but also when inhibiting the usage of long-chain fatty acids and glutamine. Intriguingly, QNS120 showed no increased dependency on mitochondrial respiration when treated with melatonin (Figure 2h). We finally evaluated the effects of melatonin treatment on ATP turnover and found that melatonin treatment depleted ATP in both cell lines (Figure 2i and Figure S2e).

In summary, we observed that melatonin treatment disrupted mitochondrial metabolism in a differential manner but increased ROS and depleted ATP in both GBM cell lines.

### 3.3. Melatonin Disrupts pH Balance in GBM while Downregulating Glycolysis

Since we observed diverse effects of melatonin over mitochondrial respiration but similar ATP depletion and ROS generation, we wanted to see how melatonin affected glycolysis as an alternative source of energy production. First, we analyzed the expression of lactate dehydrogenase (LDH), since it catalyzes the conversion of pyruvate to lactate, the main product of glycolysis. We found that LDH expression was downregulated at aMT 3.0 mM for both cell lines (Figure 3a,b). Accordingly, with this reduction in LDH expression, we found a deceleration in the production of lactate, which remained invariable at aMT 3.0 mM during the full treatment, while the vehicle and 1.5 mM lactate levels increased over time (Figure 3c). Moreover, we investigated the expression of lactate symporter MCT4 and found that MCT4 expression was reduced at aMT 3.0 mM (Figure 3d,e), correlating with the decrease in intracellular lactate production. Since the MCT4 symporter moves both lactate and protons simultaneously, these results prompted us to investigate the effects of aMT on cellular pH regulation. We found that melatonin increased the intracellular acidity (Figure 3f and Figure S3a,b) with its peak at 96 h, while extracellular pH (epH) (Figure 3g) remained more alkaline compared to the vehicle and aMT 1.5 mM. The effect on the epH was stronger in GBM1A, while in QNS120, the trend was not significant. Since we renewed media with the treatment at 48 h, we observed a minor increase between 48 and 72 h but it kept dropping after 72 h. Moreover, we analyzed other key upstream glycolytic enzymes such as pyruvate kinase subtype M2 -PKM2- (Figure 3a,h), and glyceraldehyde-3-phosphate dehydrogenase -GAPDH- (Figure 3a,i) and we found the expression of these enzymes significantly decreased after 96 h of treatment with aMT 3.0 mM for both GBM1A and QNS120. Finally, we observed a reduction in the glucose uptake overtime induced by aMT 3.0 mM for both cell lines (Figure 3j).

In summary, melatonin treatment induced a pH reversal with intracellular acidosis parallel to a downregulation in glycolysis in GBM.

### 3.4. Intratumoral Melatonin Treatment Decreases Tumor Growth In Vivo

Because the preceding experiments showed oncostatic effects caused by metabolic changes after treatment with melatonin alone or in combination with the standard of care treatments in vitro, we next investigated the effects of melatonin in vivo. We injected GBM1A GFP-Luc+ cells in athymic nude mice (flank xenograft model) and treated the mice intratumorally with melatonin or the vehicle for 2 weeks (5 days/week).

**Figure 4:**
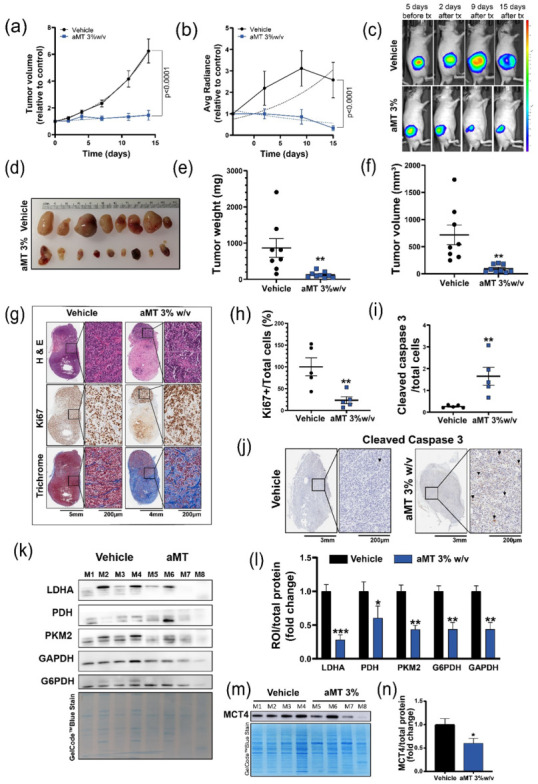
Intratumoral melatonin treatment decreases tumor growth in vivo. (**a**) Nonlinear fit exponential growth curves show caliper-measured volume or (**b**) or bioluminescence (BLI) signal of xenograft tumors treated with vehicle (*n* = 8) or melatonin 3% weight/volume (*w*/*v*) (*n* = 9), relative to day 0 (*p* < 0.001 for both; exponential growth equation rate constant k was different for each data set); (**c**) most representative BLI images before and after treatment (tx). Radiance scale in p/s/cm^2^/sr; (**d**) Photograph of extracted tumors at the treatment end point. (**e**) Tumor weight and (**f**) tumor volume at the treatment end point (*p* < 0.01 for both). (**g**) Representative histologic images of hematoxylin-eosin (H&E) staining for general morphology, Ki67 immunocytochemistry for proliferation, and trichrome staining for fibrosis; (**h**) Cell proliferation and (**i**) apoptosis were assessed by quantifying Ki67+ cells or cleaved caspase-3+ cells, respectively, and dividing by the total cell number (*p* < 0.01 for both, *n* = 5); (**j**) most representative pictures of cleaved caspase-3 immunohistochemistry (arrows point the positive cells). (**k**) Representative Western blot of LDH, PDH, PKM2, G6PDH, and GAPDH in vivo. (**l**) Western blot quantification of LDHA, PKM2, PDH, G6PDH, and GAPDH expression in the vehicle- and melatonin-treated xenografts. ROI indicates region of interest. (**m**) Representative Western blot of MCT4 in vivo; (**n**) Western blot quantification of MCT4 expression in the vehicle- and melatonin-treated xenografts. * *p* < 0.05; ** *p* < 0.01; *** *p* < 0.001.

Tumor growth was assessed by volume measurements (Figure 4a) and BLI (Figure 4b,c and Figure S4a), and both showed significantly reduced growth rates after intratumoral melatonin treatment. At the 2-week end point (Figure 4d), the tumor weight (Figure 4e) and volume (Figure 4f) were significantly reduced with melatonin treatment compared with the controls. Hematoxylin and eosin staining confirmed that the tumors had a morphologic structure that was compatible with GBM, and the Ki67 proliferation marker was quantified and shown to be significantly lower in mice treated with melatonin (Figure 4g,h). An increase in apoptosis was corroborated by cleaved caspase-3 immunostaining (Figure 4i,j). Trichrome staining showed greater fibrosis in the tumors receiving melatonin treatment compared with the controls (Figure 4g), although fibrosis did not increase in healthy tissue treated with melatonin (Figure S4b). Finally, to corroborate the melatonin effects on tumoral metabolism observed in vitro, we analyzed the protein levels of LDH, PKM2, PDH, G6PDH, GAPDH, and MCT4 in the tumors (Figure 4k–n) and showed that all were downregulated in the tumors treated with melatonin compared with the controls, as previously shown in vitro.

In summary, intratumoral melatonin induced metabolic changes that decreased tumor growth in vivo.

## 4. Discussion

GBM is considered primarily as a high-glycolytic tumor. GBM’s elevated recurrence rate is partly due to its metabolic plasticity, which allows the tumors to recalibrate their cellular demands in accordance with the bioavailability of resources in the microenvironment [21]. Consequently, GBM metabolism arises as a therapeutic target that is yet to be fully explored or pharmacologically exploited.

Previous studies of melatonin in GBM have reported the effects of this indolamine in reducing GBM stemness and viability [22,23], but none have focused on melatonin’s impact on GBM metabolism. Most tumors including GBM undergo tumor pH inversion as the intracellular media are slightly more alkaline than the microenvironment [24]. We found that melatonin is able to revert pH in GBM, inducing deleterious changes in the tumoral cells. As a consequence of this acidosis, melatonin deprives GBM of energy sources, leading to ATP depletion (Figure 2i), thereby enforcing the metabolic catastrophe [25,26,27]. To our knowledge, this study is the first to describe melatonin’s role as a pH modulator at the crossroads between intracellular acidosis (Figure 3f) and ROS production (Figure 2f).

Melatonin-induced switch-off glycolytic function (decreased glucose uptake, and LDHA, MCT4 expression) has been partially described in prostate cancer [28] and in Ewing sarcoma [29]. Given our results and accordingly to Hu and colleagues [30,31], we believe that a reduction in the aforementioned parameters would be induced by the cytosol protonation. Indeed, low pH alters the amino acid charge, thereby changing the enzymatic conformation and function [32,33]. In this context, we saw a decrease in GAPDH protein levels (Figure 3i), which has been associated with a lower glycolytic rate [30] and a decrease in breast tumor proliferation [3,34]. We observed similar effects on PKM2 (Figure 3h), which catalyzes the last step of glycolysis by converting phosphoenolpyruvate to pyruvate. Low intracellular pH is reported to reduce this enzyme activity [35]. Importantly, PDH, which catalyzes the conversion of pyruvate to acetyl Co-A and therefore its eventual entry into OXPHOS, also showed reduced expression in acidic conditions (Figure 2d) [36]. In contrast to what was reported in other tumor types [13], we observed that the mitochondrial pathway is not enough to explain the cytotoxic effects of melatonin. Moreover, enhanced ROS levels were not necessarily generated in mitochondria. For instance, in QNS120, we saw an increase in ROS without a rise in OCR. Instead, ROS increase might be explained by a decrease in the NADH turnover into NAD due to the reduction in LDHA expression (Figure 3b) [35]. Remarkably, GBM1A, the most glycolytic cell line, was more sensitive to melatonin than QNS120.

ROS levels slowed down the proliferation (Figure 1a) as well as induced cell cycle arrest at G1 or G2/M (Figure 1b) [37]. To continue from G2/M to the S phase, cells also need alkaline pH, so G2 arrest was strengthened by the cytosolic acidity [38]. Notably, intracellular acidosis, ROS, and activation of caspase 3 are critical components of the first steps of apoptosis (Figure 1c) [39]. Furthermore, we observed that melatonin diminished GBM clonogenicity and stemness (Figure 1d,e), despite the lower pH, in contrast to findings from Liu and collaborators [32]. This reduction is likely to be explained by the high ROS levels [37,40]. Likewise, the synergy observed between melatonin treatment and TMZ or RT could also be determined by the combination of redox and cytosolic acidity, consistent with previous reports [41,42]. In fact, chemoresistance is directly related to increased mitochondrial coupling and decreased ROS levels [43], while TMZ showed improved alkylating activity under acidic conditions [44]. All of these data and evidence in the literature [45] suggest a synergistic effect of acidic stress and ROS as inducers of cell death, with intracellular pH dysregulation the driving force for cytotoxic melatonin effects on GBM [3,46].

Consistent with our in vitro findings, our in vivo studies showed that local treatment with melatonin at high concentrations induced metabolic changes (decrease expression of LDH, PDH, PKM2, G6PDH, GAPDH, and MCT4). Moreover, melatonin treatment reduced tumor growth and increased apoptosis and tissue fibrosis in the tumor, but not in healthy tissues (Figure S4b), making melatonin an effective approach for GBM (Figure 4).

## 5. Limitations of This Study and Future Directions

We are aware of the limitations of the subcutaneous xenograft model, especially for brain tumors. Further research with intracranial models is needed. Clinical management of GBM usually includes tumor resection; therefore, intratumoral administration of melatonin during this treatment window could overcome the blood–brain barrier and washout of the indolamine by the liver. Our future work will include the use of intracranial models to decipher how to deliver melatonin locally to the tumor at the appropriate concentrations and in an innovative and time-sustainable manner.

Finally, we must consider the causes for the melatonin-induced cytosolic acidosis. We hypothesize that the reduction in MCT4 and LDHA are the origin of the pH imbalance caused by melatonin. However other proton transporters such as sodium/proton exchanger 1 (NHE1) or carbonic anhydrase IX (CAIX) that contribute to the movement of protons across the membrane should be considered in further research. Future rescue and loss-of-function assays are necessary to confirm the cause–effect of this correlation.

## 6. Conclusions

**Figure 5:**
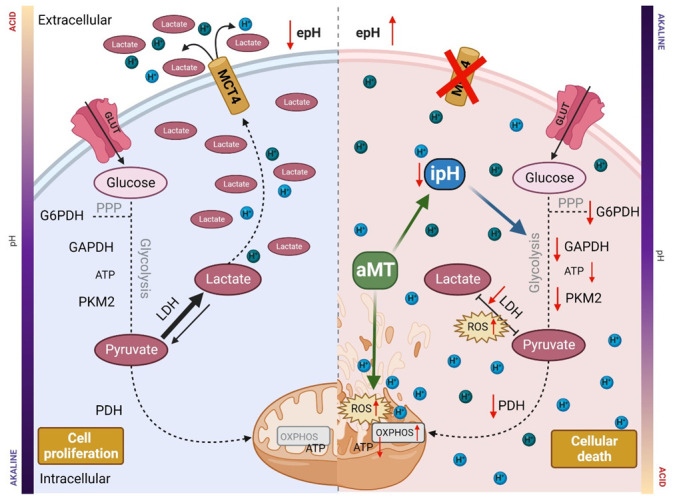
Graphical abstract. Melatonin oncostatic effects in GBM. aMT: melatonin; iPH: intracellular pH; ROS: reactive oxygen species; H^+^: proton; LDH: lactate dehydrogenase; PDH: pyruvate dehydrogenase; PKM2: pyruvate kinase; GAPDH: glyceraldehyde-3-phosphate dehydrogenase; G6PDH: glucose-6-phosphate dehydrogenase; PPP: pentose phosphate pathway, MCT4: monocarboxylate transporter 4; GLUT: glucose transporters.

Melatonin effectively decreased the proliferation of GBM cells in vitro and in vivo and caused cell death. All of these effects were likely due to a melatonin-induced metabolic catastrophe that was at least in part triggered by reduced intracellular pH and increased oxidative damage (Figure 5). Thus, pH regulation arises as a new plausible mechanism that could explain melatonin’s effects on tumoral cells and invites exploration of new therapeutic avenues.

## Figures and Tables

**Table 1:** Patient-derived cell lines.

Cell Line	Sex	Age	Molecular Subtype
GBM1A	M	-	Classical/Proneural
QNS120	M	59	Classical
GBM612	F	56	Proneural
GBM965	F	61	Classical
QNS108	M	63	Classical

## Data Availability

Not applicable.

## Supplementary Materials

The following supporting information can be downloaded at: https://www.mdpi.com/article/10.3390/cells11213467/s1, Figure S1: Metabolic baseline characterization, treatments with melatonin agonists, and combina-tion treatments of melatonin (aMT) and the standard of care for GBM; Figure S2: Seahorse com-plementary analyses; Figure S3: pH fluctuations; Figure S4: In vivo results after melatonin intra-tumoral treatment.
