# Supplementary material for: Melatonin Treatment Triggers Metabolic and Intracellular pH Imbalance in Glioblastoma
Source: Cells. 2022 Nov 2;11(21):3467. doi: 10.3390/cells11213467 (PMC9654239; doi:10.3390/cells11213467)
Supplement: Supplementary file 1 [file cells-11-03467-s001.zip › cells-1899821-supplementary.pdf]

Supplementary Materials:

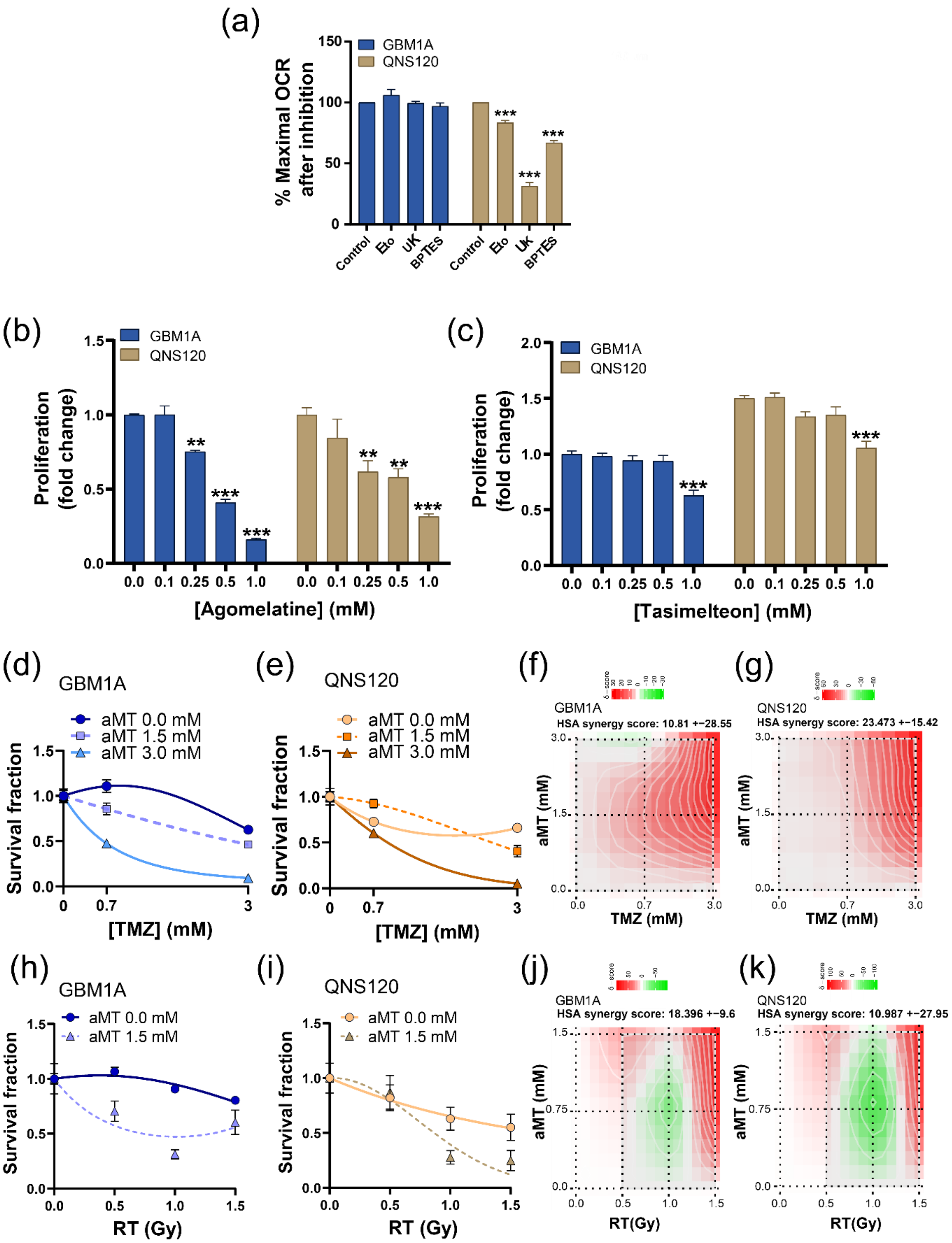

Figure S1. Metabolic baseline characterization, treatments with melatonin agonists, and combination treatments of melatonin (aMT) and the standard of care for GBM. (**a**) Percentage of maximal OCR for untreated GBM1A and QNS120 after the inhibition of carnitine palmitoyl transferase 1a (CPT1a) with etomoxir; mitochondrial pyruvate carrier (MPC) with UK5099 or glutaminase

1 (GLS-1) with BPTES. (**b**) Proliferation of GBM1A and QNS120 after 96 h of treatment with vehicle or agomelatine. (**c**) Proliferation of GBM1A and QNS120 after 96 h of treatment with vehicle or tasimelteon. (**d**) Nonlinear fit exponential growth of the combination of vehicle or aMT with TMZ in GBM1A and (**e**) QNS120. (**f**,**g**) Corresponding HSA synergy analysis. (**h**) Combination of vehicle or aMT with radiation in GBM1A and (**i**) QNS120; (**j**,**k**) corresponding HSA synergy analysis. * $p < 0.05$; ** $p < 0.01$; *** $p < 0.001$

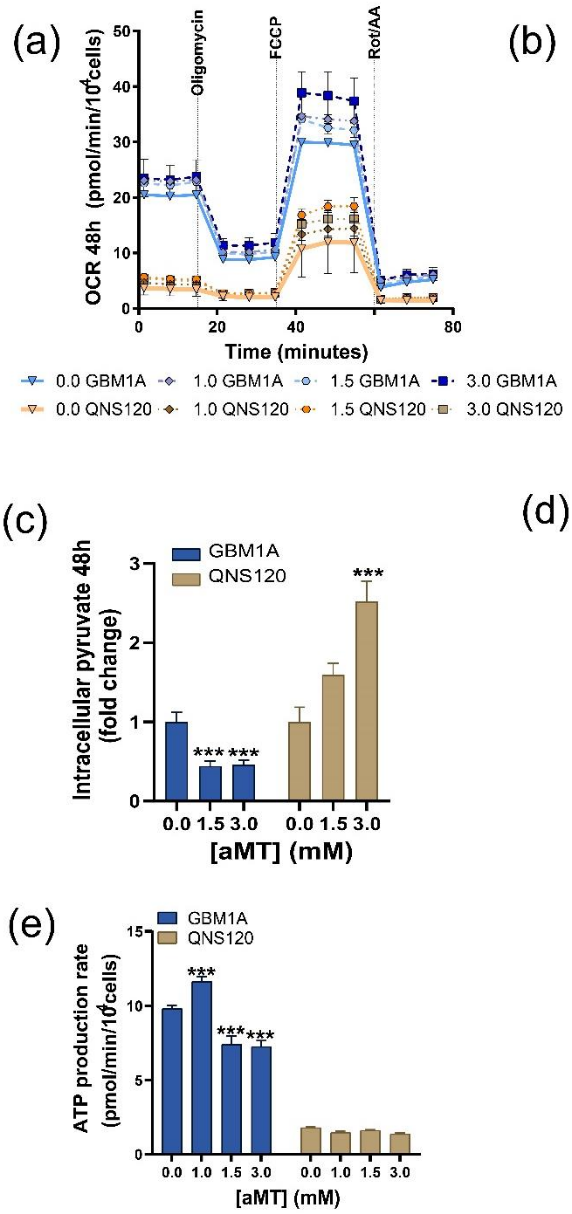

Figure S2. Seahorse complementary analyses (**a**) Oxygen consumption rate (OCR) kinetics and (**b**) basal OCR for GBM1A and QNS120 after 48 h of treatment with the vehicle or aMT. (**c**) Intracellular pyruvate in GBM1A and QNS120 after 48 h of treatment with the vehicle or aMT. (**d**) Percentage of coupling efficiency for GBM1A and QNS120 after 96 h of treatment with the vehicle or aMT. (**e**) ATP production rate from OXPHOS. * $p < 0.05$; ** $p < 0.01$; *** $p < 0.001$

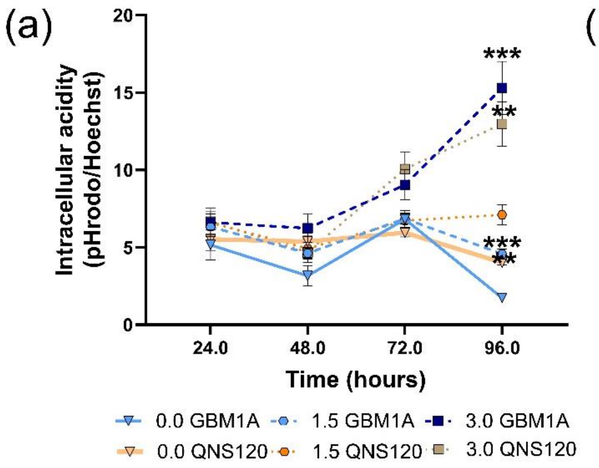

Figure S3. pH fluctuations. (**a**) Intracellular acidity over time after treatment with the vehicle or aMT in GBM1A and QNS120. (**b**) Intracellular pH (Abcam assay) of GBM1A and QNS120 after 96 h of treatment with the vehicle or aMT. RFU indicates relative fluorescent units.

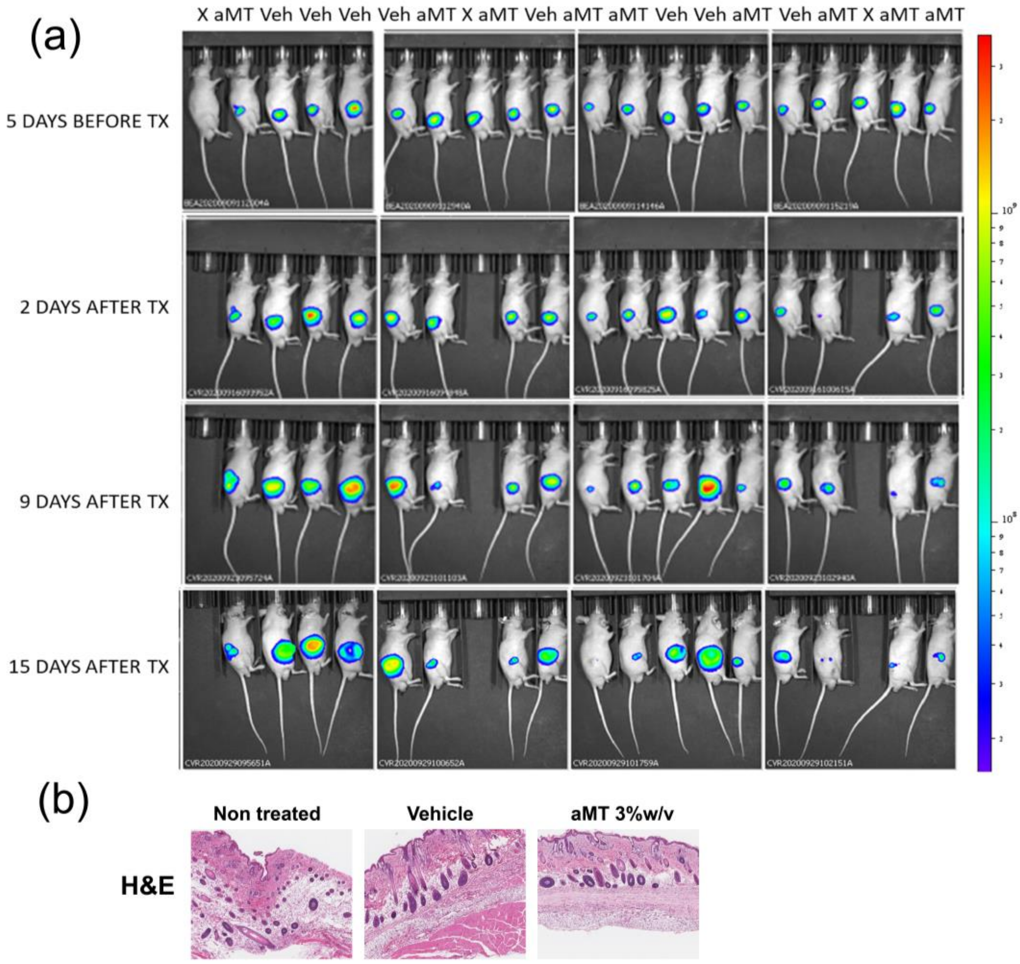

Figure S4. In vivo results after melatonin intratumoral treatment. (**a**) Weekly bioluminescence (BLI) before and after treatment (tx). Radiance scale in p/sec/cm^2/sr. Color scale min= 1.53x10^7, Max=3.78x10^9;; (**b**) hematoxylin-eosin

staining of the subcutaneous portion of skin. Non-treated or treated with vehicle or melatonin 3% *w/v* for 21 days.
